# Supplementary material for: Interacting with Members of the Public to Discuss the Impact of Food Choices on Climate Change—Experiences from Two UK Public Engagement Events
Source: Sustainability. Author manuscript; Available in PMC 2020 Jun 4. (PMC7272219; doi:10.3390/su12062323)
Supplement: Appendices [file EMS86259-supplement-Appendices.pdf]

Appendix A. Qualitative Evaluation Questionnaire (QEQ)—Long-Form Evaluation Survey

After visiting the exhibit, selected random individuals were invited to complete a survey, comprising four open questions that gauged the person's interests and perceptions of food choices and attitudes towards climate change. The aim of this survey was to evaluate the impact of the proposed activities in the stand. At the RSSSE the responses were collected via Google Docs Form (using tablets) typed by the visitors and sent online. At the Bluedot Festival, questionnaires were printed, and visitors were able to handwrite their responses, and after the event the responses were typed in a Google docs Form and the database analyzed as follows.

The questions were:

1. Have you learned anything from our exhibit? If so, what are the main things you remember?
2. Is there anything you particularly liked/disliked about the exhibit? Would you tell us more?
3. Are you likely to change any behaviours after seeing our exhibit? If so, what might you change?

4. Would you find it useful to have any tools to help you make low greenhouse gas emission's food choices? If so, what would you want from them?'

It is worth noting that, in general, the subjects presented in the responses were mentioned by an expert communicator engaging with a visitor.

Appendix B. Online Multiple-choice Survey (OMS)—Multiple Choice Survey

1. How old are you?

Under 18
18–24
25–34
35–44
45–54
55–64
65+

2. How would you describe your current diet?

I frequently eat meat and I am not interested in trying vegetarian food
I often eat meat and I occasionally eat vegetarian food
I often eat both meat and vegetarian food
I mostly eat vegetarian food and occasionally eat meat
I eat fish dairy and eggs in addition to products derived from plants
I eat dairy and eggs in addition to products derived from plants
I eat dairy in addition to products derived from plants
I eat eggs in addition to products derived from plants
I mostly only eat products derived from plants
I only eat products derived from plants

3. For what reasons do you currently choose to follow this diet? (Tick all that apply)

Environmental concerns
Animal welfare concerns
Health concerns
Cost
Availability of food
Religious beliefs
Other

4. How often do you eat beef or lamb?

Never
Rarely
Once a month
Once a week
2–3 days a week
4–5 days a week
Every day
Don't know

5. How often do you choose lower-impact protein options (such as chicken, veggie, sausages, quorn, tofu) over higher impact options (lamb or beef) to reduce your environmental impact?

Never
Rarely
Once a month
Once a week
2–3 days a week
4–5 days a week
Every day
Don't know

6. As a result of playing this game, how likely are you to consider lower impact protein options over higher impact protein options in the future to reduce your environmental impact?

Very likely
Likely
Somewhat likely
Not very likely
Not likely at all
I already always choose low impact options

7. In the next 12 months, how likely are you to adopt a more climate friendly diet?

Very likely
Likely
Somewhat likely
Not very likely
Not likely at all
I already always choose low impact options

8. For what reason(s) would you consider adopting a more climate friendly diet? (Tick all that apply)

Religious reasons
Health reasons
Environmental reasons
Some other reason

For what reason(s) is it not likely at all that you'll adopt a more climate friendly diet? (Tick all that apply)

Religious reasons
Health reasons
Environmental reasons
I enjoy my diet and do not wish to change
I do not believe that changing my diet will make a difference to the environment
Changing my diet is too difficult
Changing my diet is expensive
I'm already trying to make my diet more sustainable in my own way
Time constraints
I will in the future

Some other reason

For what reason(s) did you adopt a more climate friendly diet? (Tick all that apply)

Religious reasons
Health reasons
Environmental reasons
Some other reason

9. (a) Before playing this game how aware were you of the environmental impact of your food choices?

Not at all aware
Slightly aware
Don't know
Aware
Extremely aware

(b) And now that you've played the game?

Not at all aware
Slightly aware
Don't know
Aware
Extremely aware

10. (a) Before playing this game how concerned were you about the environmental impact of your food choices?

Not at all concerned
Slightly concerned
Don't know
Concerned
Extremely concerned

(b) And now that you've played the game?

Not at all
Slightly concerned
Don't know
Concerned
Extremely concerned

Participants for the survey were selected using convenience sampling at the 'Take a Bite out of Climate Change' stand at the RSSSE and Bluedot festival. The option to take the survey appeared after playing the CFC game on an iPad. Some individuals were playing the game unsupervised and will have clicked the 'Take a survey' button spontaneously. Usually, a member of staff asked the person if they were willing to complete it, to encourage more responses. Not every person who played the game was asked to complete the survey because this depended on how busy the stand was, and whether the staff members felt comfortable asking them to complete the survey. Additionally, a link to the game was available on the https://takeabite.info/ webpage and the www.ggdot.org webpage shared and tweeted.

In total there have been 2572 plays of the game in the period 01/07/2019–05/08/2019. The results of the survey analysis will be valid for the people who played the CFC game up to 05/08/2019. However,

they should not be expected to represent how the population of the UK would respond after playing the game. There was a strong selection effect as 81.4% of the sample were people attending the RSSSE and Bluedot festival. Furthermore, the people electing to visit the stand may have been particularly interested in the topic of food and/ or climate change. Of those visiting only a fraction chose to play the game, and of those, only a fraction completed the survey (Table A1). These people are more likely to be interested in science than the population of the UK. Hence, the sample was not representative of the UK population.

Table A1. The number of times the CFC game was played and the number of survey responses for each event (including non-response surveys). Excluding non-response surveys, there were 356 surveys with at least one question answered.

Date	Event	Number of Games Played	Number of Survey Responses
01/07/2019–07/07/2019	RSSSE	2097	271
19/07/2019–21/07/2019	Bluedot	283	45
08/07/2019–18/07/2019<br>22/07/2019–05/08/2019	Other	192	72

Appendix C. Social Media Evaluation

Table A2 shows the engagement through Twitter®over the course of the RSSSE and Bluedot. In total, the 'Take a bite out of climate change' stand had 86 tweets. The number of visualizations, however, was much more expressive (159334). The number of engagements was 2237, whist the number of clicks in the exhibit tweets was 221. In total 173 Twitter® users retweet content about the exhibit.

Table A2. Number of interactions on social media. It shows the number of online interactions using Twitter® The take a bite out of climate change had in both events.

Number of Tweets	Impressions *	Engagements **	Link Clicks	Retweets
86	15.9334	2237	221	173

* Number of times a user saw the tweet on twitter. ** Engagement on Twitter® means how many times the particular Twitter user engaged with your content: clicks anywhere on the tweet, including retweets, replies, follows, likes, links, cards, hashtags, embedded media, username, profile photo, or tweet expansion.
